# Endothelial mineralocorticoid receptor ablation does not alter blood pressure, kidney function or renal vessel contractility

**DOI:** 10.1371/journal.pone.0193032

**Published:** 2018-02-21

**Authors:** Sidsel B. Laursen, Stine Finsen, Niels Marcussen, Susan E. Quaggin, Pernille B. L. Hansen, Henrik Dimke

**Affiliations:** 1 Department of Cardiovascular and Renal Research, Institute of Molecular Medicine, University of Southern Denmark, Odense, Denmark; 2 Department of Clinical Pathology, Odense University Hospital, Odense, Denmark; 3 Feinberg Cardiovascular Research Institute and Division of Nephrology and Hypertension, Northwestern University, Chicago, IL, United States of America; 4 Cardiovascular and Metabolic Disease, IMED Biotech Unit, AstraZeneca, Gothenburg, Sweden; University Medical Center Utrecht, NETHERLANDS

## Abstract

Aldosterone blockade confers substantial cardiovascular and renal protection. The effects of aldosterone on mineralocorticoid receptors (MR) expressed in endothelial cells (EC) within the renal vasculature have not been delineated. We hypothesized that lack of MR in EC may be protective in renal vasculature and examined this by ablating the *Nr3c2* gene in endothelial cells (EC-MR) in mice. Blood pressure, heart rate and PAH clearance were measured using indwelling catheters in conscious mice. The role of the MR in EC on contraction and relaxation was investigated in the renal artery and in perfused afferent arterioles. Urinary sodium excretion was determined by use of metabolic cages. EC-MR transgenics had markedly decreased MR expression in isolated aortic endothelial cells as compared to littermates (WT). Blood pressure and effective renal plasma flow at baseline and following AngII infusion was similar between groups. No differences in contraction and relaxation were observed between WT and EC-MR KO in isolated renal arteries during baseline or following 2 or 4 weeks of AngII infusion. The constriction or dilatations of afferent arterioles between genotypes were not different. No changes were found between the groups with respect to urinary excretion of sodium after 4 weeks of AngII infusion, or in urinary albumin excretion and kidney morphology. In conclusion, deletion of the EC-MR does not confer protection towards the development of hypertension, endothelial dysfunction of renal arteries or renal function following prolonged AngII-infusion.

## Introduction

Hypertension and chronic kidney disease (CKD) are key contributors to morbidity and mortality worldwide. In fact, arterial hypertension has been projected to affect as many as 1.56 billion individuals by the year 2025, while the prevalence of CKD currently ranges between 8–16% [1, 2]. Hypertension and CKD are major problems clinically, greatly increasing cardiovascular disease risk. As such, both hypertension and CKD associate strongly with endothelial dysfunction [3–5], an adverse predictor of cardiovascular complications. Aldosterone has been described as a key player in the pathogenesis of these disease states. Blockade of the aldosterone-binding mineralocorticoid receptor (MR) have consistently been shown to reduce blood pressure in hypertensive patients [6, 7] and slow the decline in kidney function observed in individuals suffering from CKD [8, 9]. Pharmacologic blockade of the MR has protective effects on renal function in CKD. Here, MR antagonism by spironolactone retards the development of proteinuria in CKD patients, thereby slowing the progression of the disease [9]. The MR, encoded by the *Nr3c2* gene remains central to these observations. Classically, the MR expressed in renal tubular epithelium has been considered the primary mediator of adverse cardiovascular sequelae, by facilitating aldosterone-induced hypertension. However, several observations have since altered this view, including the discovery of MR expression in a variety of cells, such as the endothelial and smooth muscle cells of conduit and resistance vessels [10–12]. Furthermore, studies in individuals with primary aldosteronism find that patients suffering from this disease have a higher likelihood of developing adverse cardiovascular events, than do patients with essential hypertension [13]. Moreover, primary aldosteronism has been shown to impair renal function more severely in comparison to patients suffering from essential hypertension [14], suggesting that hypertension alone cannot account for the damage.

Aldosterone has been shown to affect vessel function via different cellular mechanisms (reviewed in detail by [15, 16]). Rapid actions of the hormone are complex and oftentimes contradictory and include inhibition of depolarization-induced vasoconstriction in afferent arterioles [12], dose-dependent vasoconstriction in renal afferent and efferent arterioles [17, 18], acute endothelium-dependent vasodilation in cerebral and mesenteric vessels [19] and inhibition of recovery from depolarization-induced vasoconstriction in mesenteric arteries [20]. Finally, different cellular mechanisms may be involved in driving the detrimental effects of aldosterone in pathological models. This has been studied by ablating the MR specifically in endothelial cells (EC) in targeted transgenic strains, subjected to chronic models of hypertension and endothelial dysfunction [21–23]. Overall, the conclusions from these studies suggest that some vasculature isolated from mice deficient in EC MR (EC-MR KO) may show altered vascular effects, however these alterations remain highly dependent on the vessels type studied [21–23]. Most experimental pathological models studied in EC-MR KO mice, show protection conferred by the EC MR towards endothelial dysfunction (i.e. a shift in balance between relaxing and contracting factors leading to altered vessel contractility) as evaluated by impairment of acetylcholine-induced endothelial-dependent vasodilation [21–23]. Moreover, some but not all studies of vessels from EC-MR KO mice in models of hypertension or endothelial dysfunction show a decreased contractility in response to constrictors, in line with a protective effect of *Nr3c2* gene ablation in endothelial cells [21–23]. For instance, Mueller *et al*, found that the EC-MR KO show decreased constriction of the coronary arterioles specifically, in response to the thromboxane agonist U46619, which is most evident following AngII-induced hypertension [23]. Thus, the varying protective effects of MR deletion in EC’s are observed during models of hypertension or endothelial dysfunction, depends on the vessel type being investigated [21–23]. The MR is also expressed in the endothelium of various segments of the renal vasculature [10, 12]. However, the effect of MR deletion in EC on vessel reactivity and function in renal vasculature in chronic models of hypertension and endothelial dysfunction has not been investigated. Accordingly, we hypothesized that lack of MR in EC’s may be protective for the renal vasculature.

While the benefits of pharmacological MR blockade on renal function and associated cardiovascular outcomes have been well described, it remains unclear how the MR in EC influences renal conduit and resistance vessel function in basal states and pathological conditions of hypertension and endothelial dysfunction. Given the essential role of the renal vasculature in maintaining adequate renal function and hemodynamics, coupled with the well-described role of the MR in protecting vessel function in chronic disease models [12, 24, 25], we aimed to delineate in detail the effects of MR deletion in the EC upon renal vessel reactivity, blood pressure, renal hemodynamics, and Na^+^ excretion rates during various durations of angiotensin II (AngII) infusion as experimental models for hypertension and endothelial dysfunction.

## Methods

### Transgenic animal models

Mice with a floxed exon 3 in the *Nr3c2* gene encoding the MR have been described in detail previously [26] and generously provided by Professors Stefan Berger and Günther Schütz (DKFZ, Heidelberg, Germany). The MR floxed mice were backcrossed from a C57BL/6 background onto an FVB/N background for a minimum of 6 generations. Mice expressing the *Tie2*-Cre transgene (FVB-Tg(Tek-cre)2352Rwng/J) were generously provided by Professor Rong Wang (UCSF, San Francisco, USA) and has been described elsewhere [27]. Intercrosses between *Nr3c2*^flox/flox^ and *Nr3c2*^flox/flox^;*Tie2*-Cre were used to generate mice with selective *Tie2*-Cre dependent endothelial excision of the floxed *Nr3c2* gene (EC-MR KO), while *Tie2*-Cre negative littermates were designated as wildtypes (WT). To evaluate *Tie2* promoter driven Cre excision efficiency, LacZ/eGFP reporter mice (*Z/EG*) that express eGFP when Cre is active were crossed with *Tie2*-Cre transgenics mice [28]. All animal experiments were conducted in accordance with Danish Law under the animal experimental permits 2015-15-0201-00798, 2015-15-0201-00479 and 2012-15-2934-00526. Experimental procedures performed at the Toronto Center for Phenogenomics were approved by the Animal Care Committee and were conducted in accordance with guidelines established by the Canadian Council on Animal Care.

### Enrichment of endothelial cells from mouse aorta and vessel dissection

Endothelial cells were isolated essentially as described in detail previously [29]. In brief, mice were anesthetized using 100 mg/kg ketamine (Ketalar) and 10 mg/kg xylazine. The thoracic aorta was cut near the transition to the abdominal cavity and the left ventricle perfused with heparin (1000 IU/ml). A needle was inserted into the proximal part of the aorta and ligated with silk thread. After rinsing with DMEM, the opposing side was ligated and filled with DMEM containing Collagenase type II solution (2mg/ml). After incubation at 37°C for 45 minutes, the collagenase solution was rinsed out, pelleted by centrifugation and stored at -80°C for subsequent analysis.

To obtain tissue for RNA expression analysis of thoracic aorta, renal and mesenteric arteries, EC-MR KO and WT littermates were anesthetized as described above and perfused with 30 ml of PBS trough the left ventricle. After vessels isolation, the surrounding fat and connective tissue was dissected off under a stereoscopic microscope and the samples stored at -80°C pending RNA extraction.

### Measurement of blood pressure and effective renal plasma flow

Measurements of blood pressure, heart rate and para-aminohippuric acid (PAH) clearance was done as described previously with modifications [30–32]. In brief, EC-MR KO and WT littermates were anesthetized using 100 mg/kg ketamine and 10 mg/kg xylazine. Measurements of mean arterial pressure and infusion of drugs was done simultaneously by implantation of micro-renathane tip based cathethers connected to polyethylene tubing into both the femoral artery and vein, respectively. The arterial catheter was connected to a pressure transducer (Föhr Medical Instruments, Hessen, Germany) for continuous measurements of mean arterial pressure (MAP) and heart rate (HR). From there, the catheters were drawn subcutaneously and exteriorized via the neck, where they were attached to a swivel system (Instech Laboratories, PA, USA) allowing free movement of the animals during recordings in an unstressed environment. The catheters were filled with heparin solution (100 IU/ml) in isotonic glucose. For analgesic pain relief, mice received subcutaneous injections of 3.75 mg/kg Temgesic (buprenorphinum) post-operatively. After a recovery period (5 days), continuous measurements of MAP and HR were recorded using LabView data acquisition software (National Instruments, Austin, TX). Infusion of AngII was done in two sets of experiments, each containing EC-MR KO mice and WT mice. The infusion rate was 10 μl/h. One experimental setup utilized a high dose of AngII (100 ng/kg/min intravenously) and another with a lower dose (60 ng/kg/min intravenously). AngII infusions commenced after a period of measuring baseline MAP and HR and until the end of the experiment.

PAH clearance was measured at baseline and during the last 24hrs of AngII infusion in the experimental group of EC-MR KO mice and WT mice receiving 60 ng AngII/kg/min to estimate effective renal plasma flow. 20% PAH (Merck) in saline was infused continuously for 24 hours allowing the clearance to reach steady state before a blood sample (100 μl) was drawn. Blood samples were collected in EDTA tubes and plasma was isolated by centrifugation. Measurements of PAH in plasma were done by a colorimetric reaction with dimethylaminocinnamaldehyde solution in an acidic environment. After 15–30 minutes of incubation, the intensity of the light was measured at 545 nm. Renal plasma flow was calculated as the infusion rate of PAH divided by the plasma concentration of PAH at steady state [32].

### Implantation of osmotic minipumps and AngII infusion

AngII (A9525, Sigma-Aldrich) was infused at a dose of 1000 ng/kg/min using osmotic minipumps (#2004, Alzet, Cupertino, CA, USA) for up to 4 weeks. The pumps were implanted subcutaneously under isoflurane inhalation anesthesia and post-operative pain relief was given in the form of Temgesic (3.75 mg/kg).

### Isometric force measurements of the renal artery

The role of the MR in EC on contraction and relaxation was investigated in the renal artery of EC-MR KO mice and WT mice at baseline or after 2 or 4 weeks of AngII infusion. Animals were subjected to continuous infusion of AngII using osmotic minipumps as described above. Mice were killed by cervical dislocation. Renal arteries were isolated under microscope and mounted in a Mulvany wire myograph (model 610M, Danish Myo Technology, Aarhus, Denmark) to measure isometric force (LabChart, AD Instruments, Colorado Springs, CO, USA). The renal arteries were incubated in physiologic saline solution (PSS, NaCl 115 mM, NaHCO_3_ 25 mM, MgSO_4_ 1.2 mM, K_2_HPO_4_ 2.5 mM, CaCl_2_ 1.3 mM, glucose 5.5 mM, and HEPES 10 mM) at 37°C, which was buffered with 5% CO_2_ in air and allowed to equilibrate for 20 minutes before experiments began. Viability of the vascular smooth muscle and endothelium was tested with phenylephrine (10^−5^ M) and acetylcholine (10^−7^ M), respectively. KCl (60 mM) was used to examine the maximal contraction, and phentolamine (10^−5^ M) was added 5 minutes prior to and during KCl to prevent depolarization induced by α-adrenergic activity. Contractility of the renal artery was determined using increasing concentrations of the thromboxane analog U46619 (starting from 2x10^-9^ M to 10^−8^ M). The endothelium-derived relaxation was investigated by precontraction of the arteries to 70% of the maximal contraction with U46619, and thereafter relaxing the arteries with increasing concentrations of acetylcholine (from 10^−9^ M to 10^−6^ M). Endothelium-independent relaxation was tested by contracting the arteries to 70% of the maximal contraction with U46619, and thereafter relaxing the arteries with increasing concentrations of sodium nitroprusside, a NO donor (10^−8^ M to 10^−5^ M). Percent relaxations to acetylcholine and sodium nitroprusside were calculated, respectively, by:

$$\frac{{\Delta Force}_{\left[Ach\right]}}{{\Delta Force}_{70\%\;contraction}}∙100\%$$

$$\frac{{\Delta Force}_{\left[SNP\right]}}{{\Delta Force}_{70\%\;contraction}}∙100\%$$

### Determination of contractile changes in afferent arterioles

After cervical dislocation, kidneys from EC-MR KO mice and WT littermates were removed and cortical afferent arterioles isolated by microdissection as described in detail previously [33]. In brief, the isolated arteriole was placed in a temperature-controlled chamber (Warner) set at 37°C covered with DMEM-F-12 media with 0.1% BSA and maintained at 5% CO2 in air. Perfusion of the arteriole was accomplished by placement of the vessel inside a holding pipette and subsequent cannulation of the arteriole by insertion of a perfusion pipette. The arteriole was opened by applying increasing perfusion pressure. Changes in luminal diameter were recorded on an inverted microscope (Olympus) at the portion of the arteriole being most responsive to application of KCl. After equilibration, vessel viability was tested by application of a 100 mM K^+^ solution (45 mM NaCl, 70 mM KCl, 25 mM NaHCO_3_, 1.2 mM MgSO_4_, 2.5 mM K_2_HPO_4_, 1.3 mM CaCl_2_, 5.5 mM glucose, and 10 mM HEPES equilibrated with 5% CO2 in air, pH = 7.4) with 0.1% in the bath and 1% BSA in the luminal perfusate) in the presence of phentolamine (10^−5^ mol/L). The contractile response to U46619 (thromboxane analogue) was also tested in afferent arterioles. Finally the vessel was constricted to U46619 for tree minutes to ensure a stable constriction and then the endothelial response was tested with increasing concentrations of acetylcholine (10^−8^ M to 10^−5^ M). The secondary dilatory responses were calculated by the formula below, as described previously [34]:

$$\frac{\left({Diameter}_{start}-{Contration}_{maksimum})-({Diameter}_{start}-{Contration}_{60seconds}\right)}{{Diameter}_{start}-{Contraction}_{maksimum}}$$

### Metabolic cage experiments

EC-MR KO mice and WT littermates were placed individually in metabolic cages. The animals were undergoing AngII infusion by osmotic minipumps as detailed above. Measurements were made on the 24 hour urine samples and blood collected on day 28, after insertion of the minipumps. Animals were housed in a temperature-controlled environment with average ambient temperature of 24.5°C degrees and relative humidity of 50%. During the course of the experiment, mice were allowed free access to food and water. EC-MR KO mice and WT were maintained on a standard rodent diet (Altromin #1324) containing 3.3% agar and added water content in the ratio of 1:1.6 of food to water. Prior to the measurements, mice were allowed several days to acclimatize to the metabolic cages.

### Measurements of electrolytes and creatinine

Creatinine in urine and blood was determined using a colorimetric assay (ABX Pentra Creatinine 120 CP kit, Horiba ABX SAS, Montpellier, France) according to the manufacturer’s instructions. Na^+^ and K^+^ were determined by flame photometry (model IL 943, Instrumentation Laboratory, Lexington, MA). Albumin was measured according to the manufacturer’s instructions using the Mouse Albumin ELISA Quantitation kit (Bethyl Laboratories, Inc., Montgomery, USA).

### Determination of Nr3c2 gene excision and expression by PCR and semi-quantitative PCR

RNA was isolated from vascular beds or aortic endothelial cells from EC-MR KO mice and WT littermates. In brief, total RNA was extracted using TRIzol Reagent (Invitrogen, Carlsbad, CA, USA) and reverse transcribed into cDNA using iScript cDNA Synthesis Kit (Bio-Rad, Copenhagen, Denmark). RNA used for semi-quantitiative PCR analysis was subjected to DNase treatment (Fermentas) prior to cDNA synthesis. The following primes spanning exon 2–3 of the *Nr3c2* gene were utilized *Nr3c2* F: CTAGGAGAAGTGATGGGTACC and *Nr3c2* R: GAAGGTCTTGAGGATCCAGTAG and mixed with iQ SYBR Green Supermix (Bio-Rad) and run on a Stratagene Mx3000 (AH Diagnostics, Aarhus, Denmark) for semi-quantitative assessment of *Nr3c2* mRNA abundance.

Primers placed in exon 2 and exon 4 of the *Nr3c2* gene were used to evaluate *Tie2*-Cre dependent excision of the exon 3 in various vascular beds by regular PCR using the following primer sequences *Nr3c2* E2 F2: CGGTCCTAGAGTACATTCCAG, *Nr3c2* E4 R2: CTGCAGGCAGGACAGTTCTTTC. The resulting PCR products were separated on agarose gels and visualized using Gel Red-Nucleic Acid Stain (Biotium).

### Sectioning of tissue for immunohistochemical staining

Kidneys isolated from WT and EC-MR mice were immersion fixed for 3 hours in 10% formalin and stored in PBS with azide until paraffin embedding. Tissue was embedded using a Tissue-Tek Vacuum Infiltration Processor 6 (Sakura Finetek, Torrance, CA, USA). Thereafter, 2 μm sections were cut on a HM 355S Automatic Microtome (Thermo Scientific) microtome.

### Immunohistochemical staining of tissue for microscopy

Immunohistochemical staining of formalin fixed paraffin embedded tissue was done as described in detail previously [35]. Briefly, sections were rehydrated from xylene through graded ethanol solutions into water and subsequently boiled in TEG buffer (10 mM Tris, 0.5 mM EGTA, pH = 9.0) for heat induced antigen retrieval using. Endogenous peroxidases and free aldehyde groups were blocked by addition of 0.6% hydrogen peroxide (H_2_O_2_) and 50 mM NH_4_Cl into PBS followed by overnight incubation at 4°C with rabbit anti-eGFP antibodies (A6455, Invitrogen) (0.1% Triton X100 in PBS). After washing, sections were incubated with horseradish peroxidase-conjugated secondary antibodies (DakoCytomation) and visualized using the Liquid DAB+ Substrate Chromogen System (3,3'-Diaminobenzidine, K3467, DakoCytomation) or using Alexa fluorophore-conjugated secondary antibodies (Molecular Probes, Eugene, OR) for fluorescence labeling. Sections subjected to 3,3'-Diaminobenzidine dependent labeling were counterstained with hematoxylin.

For histological evaluation of changes in renal morphology following AngII-infusion, sections were rehydrated to 70% ethanol and stained with Mayers Hematoxylin before being dehydrated and mounted.

### Statistical analyses

Data are presented as mean ± SEM. T-test was used for comparison between two groups, including analysis of blood pressure and heart rate at baseline. Development of hypertension and heart rate in response to AngII infusion were analysed with two-way ANOVA with a post-hoc Bonferroni’s multiple comparisons test. Analyses of myograph experiments were done using one- or two-way ANOVA with Bonferroni’s multiple comparisons test. All tests were performed via the GraphPad Prism statistics program (version 6.0). Values were considered significant with p<0.05. In case of a skewed distribution, logarithmic values were used.

## Results

### Evaluation of *Nr3c2* excision in mouse endothelial cells

**Fig 1:**
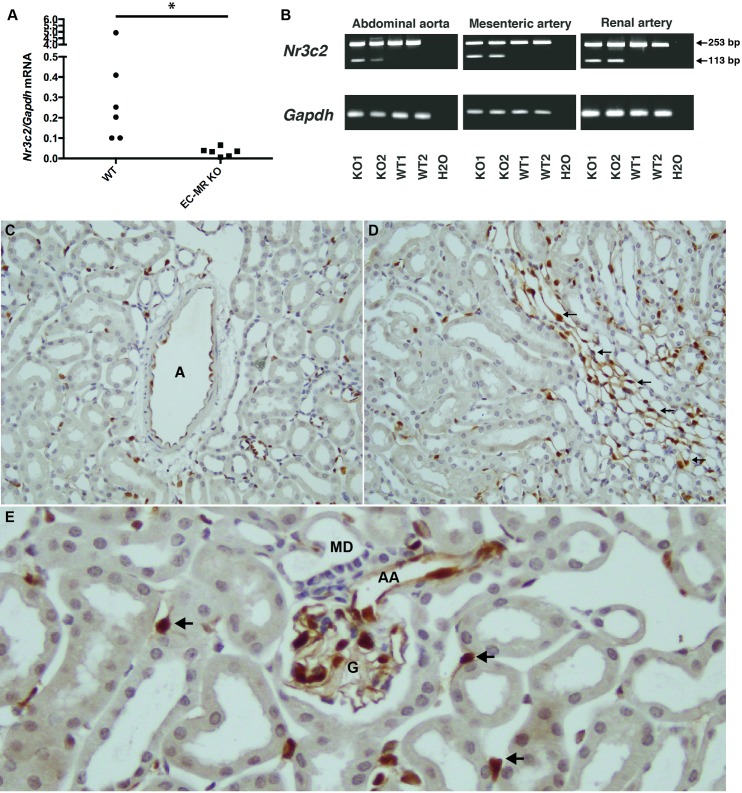
Evaluation of *Nr3c2* excision in endothelial mineralocorticoid receptor knockout mice (EC-MR KO) and in littermate controls (WT). Expression of mRNA of *Nr3c2* from isolated aortic endothelial cells in EC-MR KO and WT mice (n = 6 per group). (B) Expression of mRNA from the abdominal aorta, mesenteric artery and renal artery. Amplification of mRNA transcribed from the *Nr3c2* gene using a primer set spanning exons 2–4 would yield only one fragment of 253 bp in mice carrying the floxed *Nr3c2* alleles, whereas mice expressing both the floxed *Nr3c2* gene as well as the Cre recombinase driven by *Tie2* promoter would yield another fragment of 113 bp. (C-E) Immunohistochemical analysis of kidney from intercrosses of mice expressing Tie2-Cre and the LacZ/eGFP reporter gene (*Z/EG*). eGFP expression is detected in cells positive for Cre recombinase. (C) Cre-dependent excision allowing eGFP expression can be detected in arterioles within kidney (denoted A). (D) eGFP positive cells were also present in the renal microvasculature, in the vasa recta bundels in the outer medulla (indicated by arrows) and (E) afferent arterioles (AA) located adjacent to the macula densa (MD), in glomerular (G) endothelial cells lining the capillary loops as well as the peritubular capillaries (indicated by arrows).

To validate that expression of the *Nr3c2* gene encoding the MR was reduced in endothelial cells of EC-MR KO mice, we isolated endothelial cells from the aorta of EC-MR KO and WT littermates. Expression analysis of *Nr3c2* in endothelial isolates as evaluated by semi-quantitative PCR, revealed a significantly reduced expression of *Nr3c2* gene in samples obtained from EC-MR KO mice (Fig 1A). To investigate in detail whether Cre activity would lead to deletion of the *Nr3c2* gene in select vascular beds, we isolated thoracic aortas, renal arteries, and mesenteric arteries from EC-MR KO and WT mice after perfusion with phosphate buffered saline (Fig 1B). Given that exon 3 of the *Nr3c2* is floxed in this model [26], PCR amplification of mRNA transcribed from the *Nr3c2* gene, using a primer set spanning exons 2–4, would yield a single fragment corresponding in size to 253 bp in both *Nr3c2* floxed mice not expressing the *Tie2*-Cre driven recombinase and *Nr3c2* floxed mice expressing the *Tie2*-Cre transgene. Furthermore, if the given endothelial cell expresses the floxed *Nr3c2* gene as well as the Cre recombinase driven by *Tie2*, amplification of another shorter mRNA product lacking exon 3, corresponding in size to 113 bp will also be present in these animals (Fig 1B). As can be observed, a shorter fragment could be identified in all vessels investigated only in the EC-MR KO mice (Fig 1B).

In an effort to determine which subpopulations of endothelial cells in kidney were expressing the *Tie2*-Cre recombinase and hence able to inactivate the floxed *Nr3c2* gene, the *Tie2*-Cre strain was intercrossed with *Z/EG* reporter mice. The *Z/EG* mouse expresses eGFP when sufficient Cre activity is present in the cell. Immunohistochemical analysis showed eGFP expressing cells in all endothelial compartments within kidney, ranging from larger renal vessels, afferent arterioles, peritubular capillaries to vasa recta bundles (Fig 1C–1E), suggesting that if an endothelial cell express the *Nr3c2* gene product, Cre recombinase mediated knockout is likely to occur.

### Measurements of mean arterial pressure in EC-MR KO and WT mice

**Fig 2:**
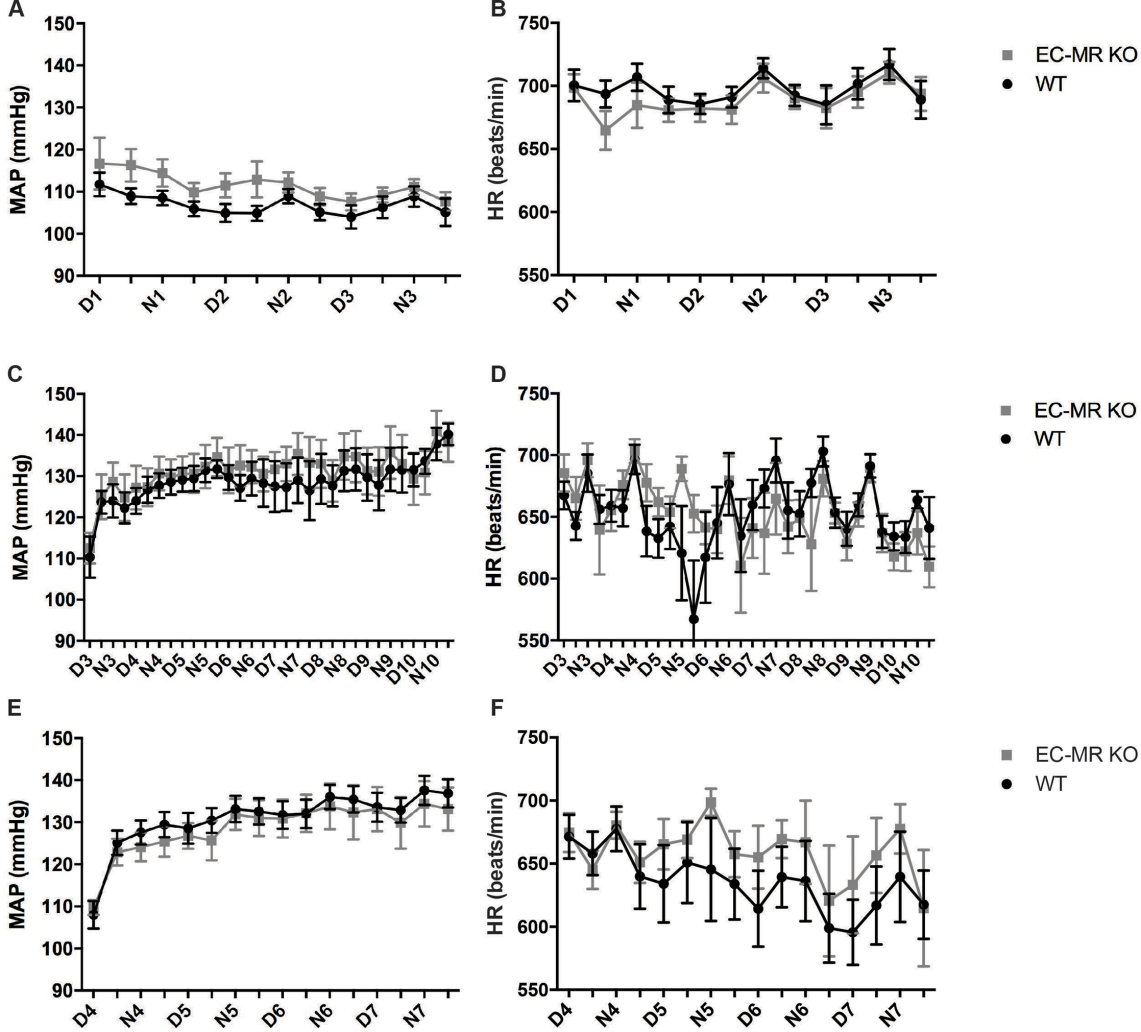
Evaluation of mean arterial pressure (MAP) and heart rate (HR) between wildtype WT and EC-MR KO during baseline and AngII infusion. Baseline (A) MAP and (B) HR were measured (D1-N3) in EC-MR KO and WT mice (n = 14–18 per group). (C) MAP and (D) HR were measured for 7 days (D3-D10) after start of AngII infusion (60 ng/kg/min) (n = 7–9 per group). (E) MAP and (F) HR were also measured for 4 days (D4-D7) after infusion of angiotensin II (AngII 100 ng/kg/min) (n = 7–9 per group). All data are mean ± SEM.

Mean arterial pressure (MAP) and heart hate (HR) was measured at baseline and in two experimental settings with EC-MR KO mice and WT receiving continuous intravenous infusions of AngII at dosages of 60 ng/kg/min or 100 ng/kg/min. The groups of mice receiving 100 ng/kg/min showed an increased mortality during infusion, and therefore the dosage was lowered to 60 ng/kg/min. Measurements of MAP during baseline showed no changes in blood pressure, neither when analysed by evaluation of average blood pressures (p = 0.14) nor area under curve (p = 0.49) (Fig 2A). Changes in HR may aid in stabilizing blood pressure via compensatory reductions in beat frequency, however no changes between the EC-MR KO and WT mice could be detected at baseline (Fig 2B). AngII infusion dose dependently increased MAP in both EC-MR KO and WT mice. The effect of 60 ng/kg/min AngII infusion on alterations in MAP was not different between EC-MR KO and WT mice (Fig 2C). Heart rate also did not differ between genotypes (Fig 2D). Similar findings were obtained with infusion of 100 ng/kg/min of AngII (Fig 2E & 2F).

### EC-MR deletion does not alter contractility or relaxation of the renal artery

**Fig 3:**
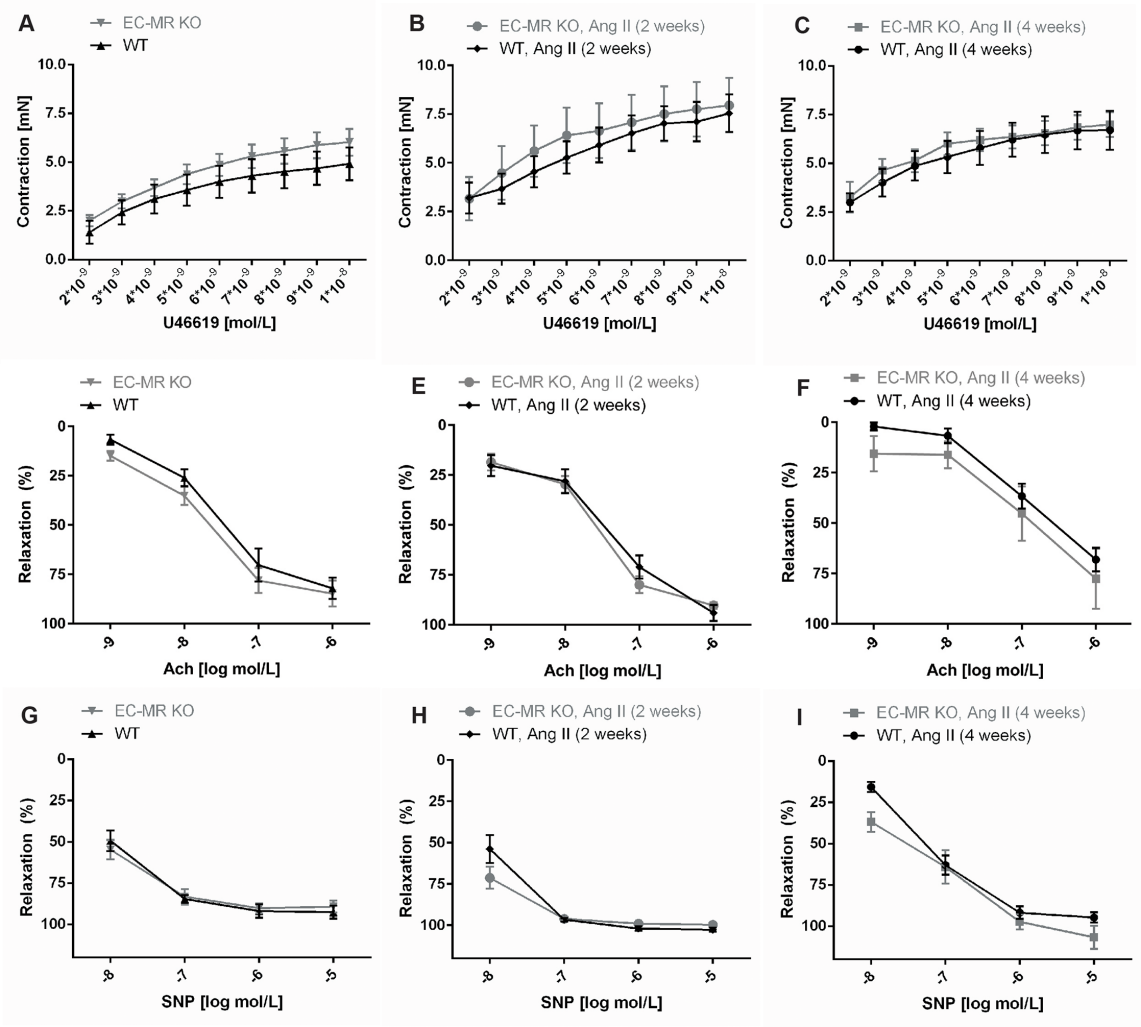
Determination of contractility and endothelial function in the renal artery in WT and EC-MR KO mice. Effect on contraction to the thromboxane analog U46619 of (A) WT and EC-MR KO at baseline (n = 8–12 per group), (B) WT and EC-MR KO after 2 weeks of angiotensin II (AngII) infusion (n = 7–8 per group) and (C) WT and EC-MR KO after 4 weeks of AngII infusion (n = 11–7 per group). Effect on acetylcholine (Ach)-induced endothelial-dependent relaxation of renal arteries from WT and EC-MR KO at baseline (n = 7–11 per group), (E) WT and EC-MR KO after 2 weeks of AngII infusion (n = 7–8 per group), and (F) WT and EC-MR KO after 4 weeks of AngII infusion (n = 11–7 per group). Effect on endothelial-independent relaxation by the NO donor, sodium nitroprusside (SNP) in (G) WT and EC-MR KO at baseline (n = 7–11 per group), (H) WT and EC-MR KO after 2 weeks of AngII infusion (n = 7–8 per group), and (I) WT and EC-MR KO after 4 weeks of AngII infusion (n = 11–7 per group). All data are mean ± SEM.

Myographic recording of vessel wall tension were used to evaluate contraction and relaxation responses of the renal artery to the thromboxane analog U46619, acetylcholine and sodium nitroprusside (SNP). Renal arteries were isolated from EC-MR KO and WT mice before or after subcutaneous infusion of AngII for 2 or 4 weeks by osmotic minipumps, delivering the drug at a dose of 1000 ng/kg/min. Changes in contractility were subsequently analyzed in response to increasing concentrations of the thromboxane analog U46619. No changes in contractility could be observed after application of increasing amounts of U46619 during baseline and after 2 or 4 weeks of AngII infusion in WT mice (Fig 3). The EC-MR KO mice displayed a similar contractile response to U46619 as WT both during baseline (Fig 3A), after 2 weeks of AngII infusion (Fig 3B), and after 4 weeks of AngII infusion (Fig 3C). Endothelial-dependent vessel relaxation was measured in renal arteries from WT and EC-MR KO mice using acetylcholine. Application of increasing concentrations of acetylcholine in the presence of U46619 at the dosage leading to 70% of precontraction values, resulted in dose-dependent dilatation of the renal arteries. No change in the vasorelaxation actions of acetylcholine was observed between WT and EC-MR KO mice both at baseline or following 2 or 4 weeks of AngII infusion. Impaired endothelial-derived responses to acetylcholine (10^−7^ M) were observed after 4 weeks of AngII infusion in both genotypes, in comparison to that observed at baseline (S1 Fig), suggesting the development of endothelial dysfunction after prolonged AngII-infusion. However, there was no difference in relaxation of the renal artery between the WT and EC-MR KO at baseline (Fig 3D), after 2 weeks of AngII infusion (Fig 3E), or after 4 weeks of AngII infusion (Fig 3F). The nitric oxide (NO) donor sodium nitroprusside (SNP) was used to evaluate endothelium-independent relaxation of the renal artery. Again, contractile changes were assessed in the presence of U46619 at the dosage leading to 70% contraction of the vessel. No differences were noted in either genotype in response to SNP mediated dilatation of the renal arteries (Fig 3G–3I). In brief, these observations suggest that the MR in EC in renal arteries does not augment vessel contractility, nor play a role in development of endothelial dysfunction.

### No change in the contractility of afferent arterioles or effective renal plasma flow between EC-MR KO and WT mice

**Fig 4:**
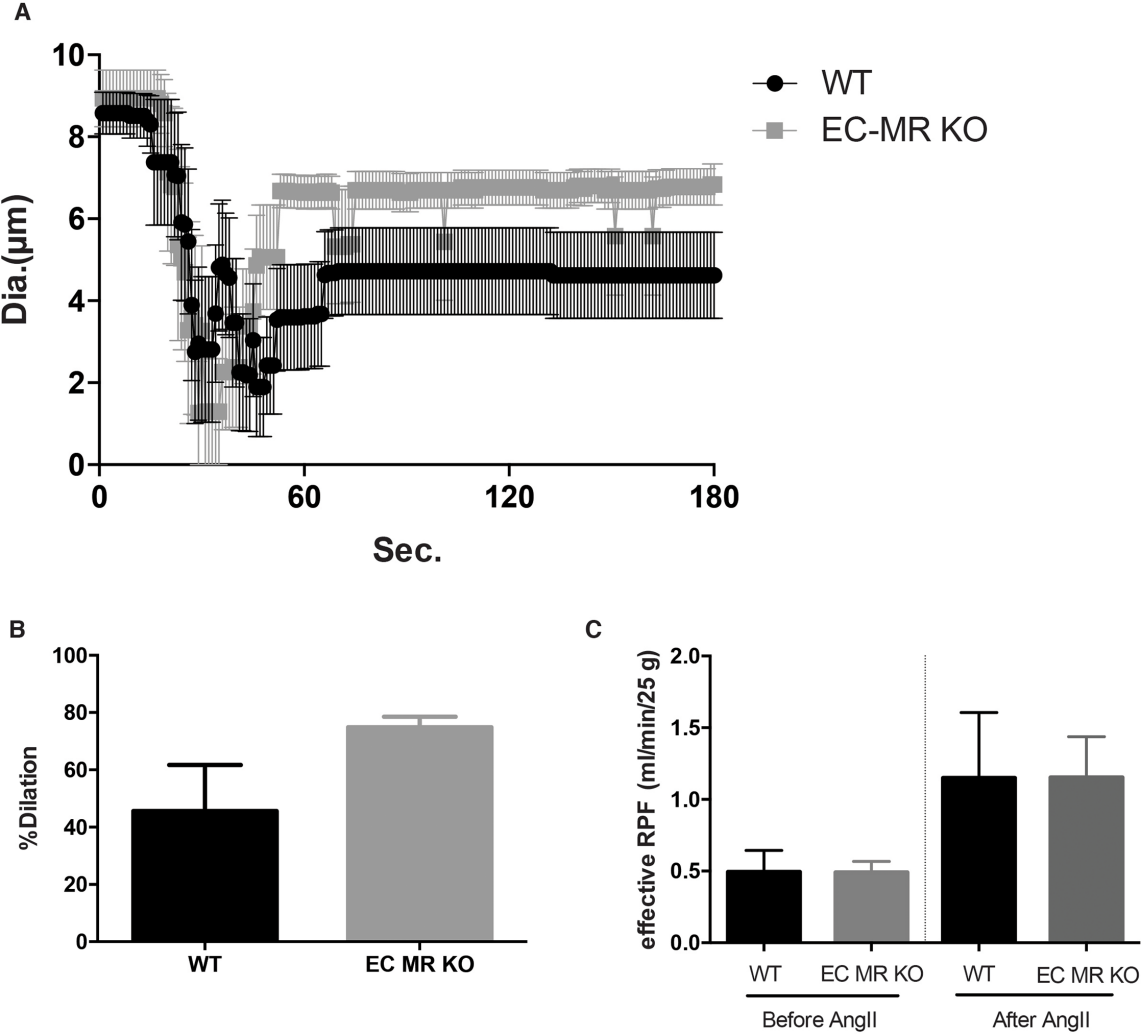
Vascular response of afferent arterioles and renal plasma flow (RPF) of WT and EC-MR KO mice. (A) Depolarized-induced vasoconstriction to 100 mM K^+^ solution of WT and EC-MR KO (n = 6–5 per group). (B) Secondary dilatory responses of afferent arterioles of WT and EC-MR KO (n = 6–5 per group). (C) Effective RPF calculated from para-aminohippurate clearance before and after 60 ng/kg/min infusion of angiotensin II (AngII) of WT and EC-MR KO (n = 5–7 per group).

Chronic aldosterone exposure has previously been shown to inhibit NO-mediated dilatation (secondary dilatation after K^+^ induced constriction) of afferent arterioles [12]. We therefore sought to evaluate whether endothelial MR deletion would affect the function of renal resistance vessels by measuring changes in luminal diameter in perfused microdissected afferent arterioles. Depolarization-induced vasoconstriction (100 mM K^+^ solution) did not show significant changes between EC-MR KO and WT mice (Fig 4A). Secondary dilatory responses also remained unaltered between the two groups of mice (Fig 4B). Moreover, no change was observed in response to increasing concentrations of acetylcholine (10^−9^ M to 10^−6^ M).

We also attempted to investigate contractile changes in afferent arterioles isolated from mice receiving AngII infusion for 2 weeks, however technically this proved difficult, due to the strong constrictor response of AngII. To evaluate whether deletion of the endothelial MR may change renal plasma flow (RPF), para-aminohippurate clearance was measured during baseline and following 7-day infusion of 60 ng/kg/min of Ang II and the effective RPF was determined. At baseline, no change could be observed in effective RPF between the EC-MR KO and WT mice (Fig 4C). Infusion of AngII resulted in a mean increase in effective RPF from 0.49 ml/min/25g mouse to a mean of 1.15 ml/min/25g mouse. No differences could be observed between the EC-MR KO and WT mice either before or after infusion of AngII (Fig 4C).

### Evaluation of renal function in mice EC-MR KO and WT mice undergoing 4 weeks of AngII infusion

**Fig 5:**
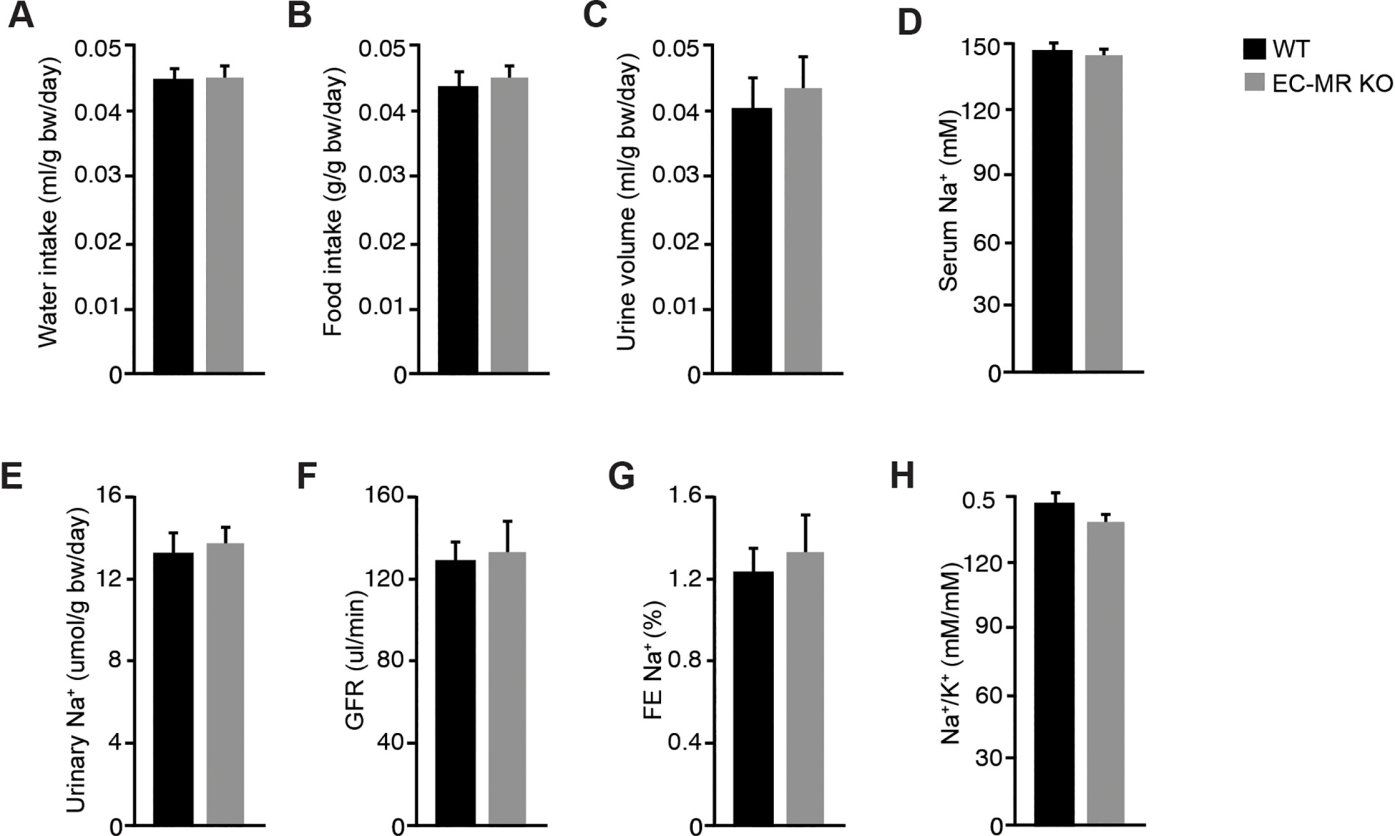
Evaluation of renal function and Na^+^ handling. Renal function was evaluated in WT and EC-MR KO after 4 weeks of angiotensin II infusion by osmotic minipumps (1000 ng/kg/min) in metabolic cages. (A) Water intake, (B) Food intake, (C) urine volume, (D) serum Na^+^ concentration, (E) Urinary Na^+^ excretion, (F) estimated glomerular filtration rate determined by creatinine clearance, (G) fractional excretion of Na^+^. (H) Urinary Na^+^/ K^+^ ratio (n = 8 per group). All data are mean ± SEM.

We also wanted to investigate, whether EC MR may elicit alterations in estimated eGFR and urinary Na^+^ rates between EC-MR KO and WT mice undergoing AngII infusion for 4 weeks. After 4 weeks of AngII infusion animals were placed in metabolic cages for acclimatization and thereafter, creatinine clearance as well as urinary Na^+^ excretion rates were measured for 24 hours. Water and food intake did not differ between EC-MR KO and WT mice (Fig 5A and 5B). Similarly, urinary volume remained unchanged (Fig 5C). Furthermore, neither serum Na^+^ nor urinary Na^+^ excretion was altered during AngII infusion between the two genotypes (Fig 5D and 5E). Finally, neither estimated GFR as determined by creatinine clearance (Fig 5F) nor the fractional excretion of Na^+^ (FENa, Fig 5G) differed between groups following 4 weeks of AngII infusion. Furthermore, No change in Na^+^/ K^+^ ratio could be detected between groups (Fig 5H).

**Fig 6:**
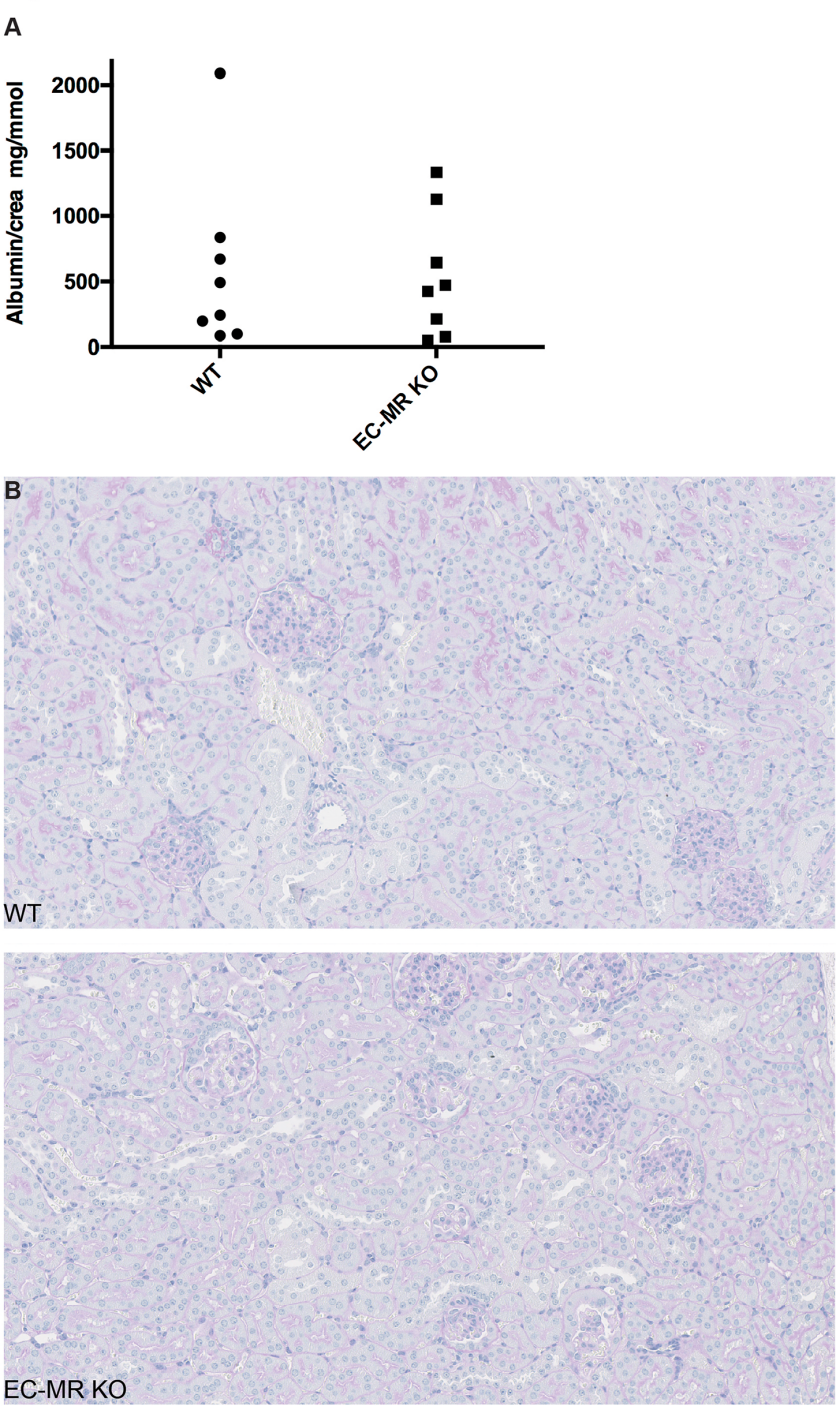
Evaluation of renal damage. Renal damage was evaluated following 4 weeks of angiotensin II (AngII) infusion by osmotic minipumps (1000 ng/kg/min) in metabolic cages. (A) Albumin/creatinine ratio between WT and EC-MR KO littermates (n = 8 per group). (B-C) Histopathological changes evaluated by PAS staining in WT and EC-MR KO mice after 4 weeks of AngII infusion. (B) Evaluation of histopathological changes between WT and EC-MR KO were determined using PAS stained kidney crossections. Overall, no marked changes in structure could be seen between genotypes.

To evaluate whether EC-MR KO mice would be protected from renal damage following 4 weeks of AngII infusion, urinary albumin excretion, tubular and glomerular histology as well as peritubular density was analyzed. No change was apparent in 24-hour urinary albumin excretion as well as in the albumin/creatinine ratio between EC-MR KO and WT mice (Fig 6A). The histopathological changes were evaluated using PAS stained sections, and changes were scarce. The overall structure of the kidneys was normal without hydronephrosis. In both groups, the glomeruli were normal without sclerosis or hypercellularity. Minor focal areas with tubular dilatation and presence of hyaline casts were noted, and some perivascular inflammation was present, mainly around extrarenal branches of the renal artery within both genotypes after AngII infusion for 4 weeks. In one KO mouse the larger arteries showed intimal thickening and some inflammation together with perivascular inflammation. One WT mouse had more chronic intimal thickening and one a few small extrarenal arteries with thrombosis. (Fig 6B and 6C).

## Discussion

The current study delineates in detail the effects of MR deletion in EC on renal vessel reactivity and renal function. Overall, our data suggest that the MR in EC does not play a role in altering contractility of the renal arteries during conditions as endothelial dysfunction, nor does it contribute to changes in renal function or hemodynamics. These conclusions can be drawn from the following observations. *i)* MR deletion in EC does not modify pressure at baseline or in response to AngII. *ii)* Renal artery vasodilatory responses to acetylcholine do not differ between EC-MR KO and WT mice, neither at baseline nor after 2 or 4 weeks of AngII infusion. *iii)* No change in contractility of afferent arterioles was noted in EC-MR KO and WT mice and *iv)* effective RPF was not affected at baseline or following AngII infusion between the genotypes. *v)* Urinary Na^+^ handling and estimated eGFR does not differ between genotypes after 4 weeks of AngII infusion. *vi*) No change in urinary albumin excretion or histopathological changes were noted between the genotypes.

Deletion of the EC MR does not affect relaxation or constriction during basal conditions in aorta and mesenteric arteries [21, 22]. Mueller *et al*, have shown that the EC-MR is involved in vasoconstriction in select vascular beds and this constriction is also dependent on the specific contractile agonist employed. Specifically, they found that the constriction of the coronary arterioles to U44619 was lower in the EC-MR KO mice in the basal state, which was not found in mesenteric arterioles [23]. This evidence points towards diverse roles for the EC MR in various vascular beds and vessel types. Heterogeneity of endothelium-dependent responses between different vascular beds are well-known (e.g. [36]). Classical examples are the response to acetylcholine in canine blood vessels where endothelium-dependent relaxations occur in most vessels tested, while endothelium-independent contractions are found in cerebral arteries [37], or the presence of endothelium-dependent relaxations to vasopressin in cerebral, but not in peripheral arteries [38]. Differences between vascular beds may rely on the amount of MR expression as well as downstream signalling mediators utilized by the MR.

Recent studies examining MR function in various vascular beds in experimental models with endothelial dysfunction, hypertension and/or high circulating aldosterone concentrations, clearly suggest that not all vascular beds show altered vessel contractility in response to deletion of the EC MR [21–23]. For instance, aldosterone- or DOCA/salt-induced endothelial dysfunction did not affect vessel contraction in the aorta and mesenteric arteries from EC-MR KO mice in comparison to WT mice [21, 22]. In contrast, AngII-induced hypertension resulted in contraction-dependent alterations in vessel function in the coronary arterioles alone, and not the mesenteric vessels [23]. The absence of protective effects on renal artery contractility upon EC MR deletion may be understood in light of these divergent findings in various vascular beds.

In the current study we found no change in baseline blood pressure between EC-MR KO and WT mice, either at baseline or following AngII infusion. The lack of change is in line with previous studies investigating the effects of EC MR deletion on blood pressure maintenance [22, 23]. Overall, these observations suggest that the EC MR does not contribute to blood pressure maintenance in basal states and following experimental hypertensive models. However, overexpression of the MR in the EC of mice does promote an increase in blood pressure [10]. Here, EC dependent overexpression of the MR promotes an increase in blood pressure, likely resulting from an exaggerated myogenic response. Therefore, it is possible that MR activation may lead to altered contractility, which could facilitate the development of hypertension. However, it is unclear whether these MR EC overexpressors inappropriately express the MR in endothelia normally devoid of the MR.

In our model, the EC-MR KO did not protect against contraction of the renal artery, neither at baseline nor after 2 or 4 weeks of AngII infusion. Furthermore, our data suggest that deletion of the MR in EC during baseline and in conditions of hypertension and endothelial dysfunction does not affect overall acetylcholine-induced endothelium dependent responses, and likely the bioavailability of NO in renal arteries. However, these responses may also differ between vascular beds. For instance, as reported by Rickard *et al*, deletion of the MR in EC was protective against the development of endothelial dysfunction as evaluated by acetylcholine-dependent endothelial responses in aorta, but not in mesentery [22]. These data suggests that the effect of the MR in the EC may act on differing hitherto unknown molecular mechanisms, depending on the vascular beds. Interestingly, mice overexpressing the MR in endothelial cells show an increased response to constrictors such as U46619 in mesenteric vessels, while no difference in endothelial-dependent acetylcholine response could be observed [10]. Moreover, most pronounced effects of EC MR deletion seems to be reported in conditions of endothelial dysfunction, rather than during basal conditions [21–23]. This may result from the observation that abundance in endothelium is much lower than renal tubular epithelial cells, suggesting the EC MR may be less responsive to physiological circulating levels of aldosterone, while primarily activated during conditions of high circulating aldosterone. However, these effects were not observed in this study of the vasculature of the renal artery.

No changes in vessel function could be observed in afferent arterioles. Being the major resistance vessel in kidney, this would play an important role in regulating renal blood flow. As such, no changes could be found in blood flow in the basal state between the genotype, suggesting that the EC MR does not contribute to regulating flow under normal physiological conditions. However, we have previously shown that aldosterone have an acute effect on afferent renal vessels, by inhibiting depolarization-induced vasoconstriction [12]. This beneficial effect occurs after application of aldosterone concentrations found in the low physiological range and is blocked by MR antagonists. Hence, multiple effects of aldosterone may modulate vessel function during various physiological and pathophysiological settings. Thus, aldosterone may exert acute stimulatory effect on vessel function, while chronically high levels would lead to the pathological effects [12]. Similar observations of acute aldosterone effects have been made in aortic rings [39]. Infusion of AngII resulted in an increase in effective renal plasma flow, while no differences could be observed between the WT and EC-MR KO mice. The increase in effective RPF following AngII infusion is in line with previous observations from Casare *et al*. who find that long term infusion of AngII increase RPF [40] and a concomitant decrease in renal vascular resistances. This increase in RPF may result from an impairment of renal auto-regulation following long-term blood pressure elevation. Given the technical difficulties we had in measuring afferent arteriole function following chronic AngII infusion in mice, we do not know whether long-term administration could affect vessel function differently, than in the EC-MR knockout mice.

Given the strong constrictor effects of AngII, one would predict that in states of endothelial dysfunction, as induced by 4 weeks of AngII infusion in our study, and in response to vasoconstrictors such as AngII itself, could yield alterations between the genotypes in urinary Na^+^ excretion and GFR. Such alterations could be elicited as modulation of renal artery resistance would alter the pressure-natriuresis relationship, and thereby impair the kidneys ability to adequately sense blood pressure akin to renal artery stenosis. Our model does not show such changes in urinary Na^+^ excretion and GFR, which could be due to a variety of reasons, but may potentially result from the strong constrictor response already elicited by infusion of supraphysiological AngII dosages, contracting the arteries of WT and EC-MR KO to the same extend *in vitro*. However, the observed differences in contractility and relaxation between different vascular beds could influence the function of the EC MR *in vivo* in conditions with endothelial dysfunction and a less potent constrictor milieu.

It is therefore speculated, that the effect of the EC MR differs depending on the type of vasculature and the organs being investigated. The role of the EC-MR in protecting against pathophysiological changes seems much more pronounced in the heart, where deletion of the EC MR attenuated development of cardiac injury. However, in the kidney, MR deletion in the EC did not affect the development of renal injury [41]. This is in line with previous studies of the aorta, where EC MR deletion protected against development of endothelial dysfunction [22, 42], while we did not observe any effect of EC MR deletion on the renal artery after 4 weeks of AngII infusion, nor any differences in albuminuria, or renal Na^+^ handling. The beneficial effect of pharmacologic antagonism of the MR on the kidney could also stem from blockade of the MR in the smooth muscle cells, rather than MR in EC. In fact, conditional deletion of the *Nr3c2* gene in smooth muscle cells protected against renal injury in an ischemia-reperfusion model, while the knockout of the MR in EC did not affect the injury [43].

This study investigated in detail the effect of the EC MR on the renal vasculature. We found no effect of EC-MR deletion on development of hypertension, development of endothelial dysfunction in the renal artery, or any alterations in RPF and afferent arteriole contractility or overall renal function and morphology. It therefore appears that the EC MR only plays a minor role in renal physiological mechanisms, nor is protective against pathological changes in kidney and renal vasculature, in models of experimental hypertension and endothelial dysfunction.

## Supporting information

S1 Fig Determination of endothelial function in the renal artery at baseline and after 4 weeks of AngII infusion in WT and EC-MR KO mice. Effect on contraction to the thromboxane analog U46619 of (A) WT and EC-MR KO at baseline (n = 8–12 per group), (B) WT and EC-MR KO after 2 weeks of angiotensin II (AngII) infusion (n = 7–8 per group) and (C) WT and EC-MR KO after 4 weeks of AngII infusion (n = 11–7 per group). Effect on acetylcholine (Ach)-induced endothelial-dependent relaxation of renal arteries from WT and EC-MR KO at baseline (n = 7–11 per group), (E) WT and EC-MR KO after 2 weeks of AngII infusion (n = 7–8 per group), and (F) WT and EC-MR KO after 4 weeks of AngII infusion (n = 11–7 per group). Effect on endothelial-independent relaxation by the NO donor, sodium nitroprusside (SNP) in (G) WT and EC-MR KO at baseline (n = 7–11 per group), (H) WT and EC-MR KO after 2 weeks of AngII infusion (n = 7–8 per group), and (I) WT and EC-MR KO after 4 weeks of AngII infusion (n = 11–7 per group). All data are mean ± SEM.
(TIFF)
