# Supplementary figures and images for: Endothelial mineralocorticoid receptor ablation does not alter blood pressure, kidney function or renal vessel contractility
Source: PLoS One. 2018 Feb 21;13(2):e0193032. doi: 10.1371/journal.pone.0193032 (PMC5821352; doi:10.1371/journal.pone.0193032)

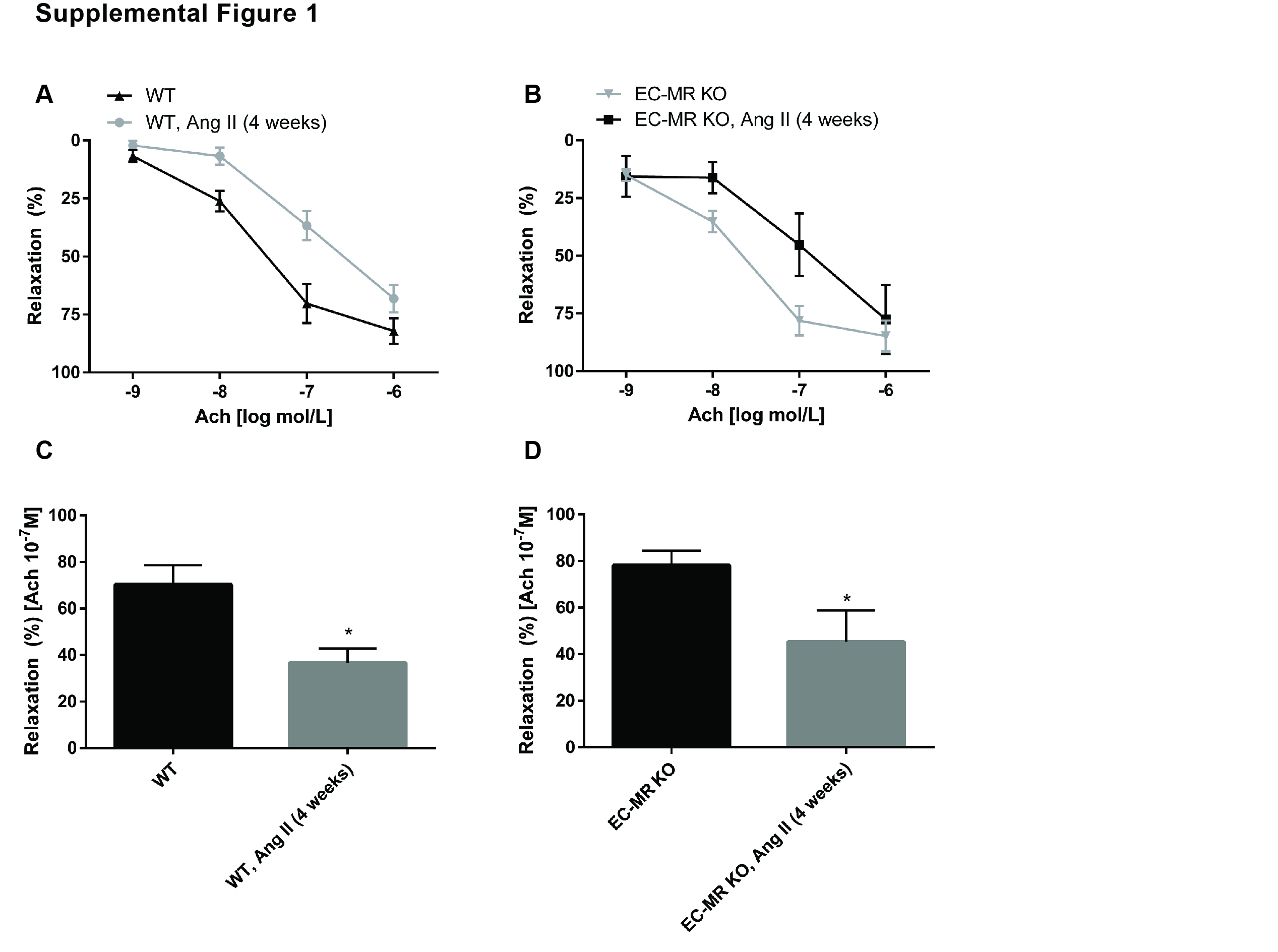

Supplement: S1 Fig — Effect on contraction to the thromboxane analog U46619 of (A) WT and EC-MR KO at baseline (n = 8–12 per group), (B) WT and EC-MR KO after 2 weeks of angiotensin II (AngII) infusion (n = 7–8 per group) and (C) WT and EC-MR KO after 4 weeks of AngII infusion (n = 11–7 per group). Effect on acetylcholine (Ach)-induced endothelial-dependent relaxation of renal arteries from WT and EC-MR KO at baseline (n = 7–11 per group), (E) WT and EC-MR KO after 2 weeks of AngII infusion (n = 7–8 per group), and (F) WT and EC-MR KO after 4 weeks of AngII infusion (n = 11–7 per group). Effect on endothelial-independent relaxation by the NO donor, sodium nitroprusside (SNP) in (G) WT and EC-MR KO at baseline (n = 7–11 per group), (H) WT and EC-MR KO after 2 weeks of AngII infusion (n = 7–8 per group), and (I) WT and EC-MR KO after 4 weeks of AngII infusion (n = 11–7 per group). All data are mean ± SEM. (TIFF) [file pone.0193032.s001.tiff]
